# Bacterial Endocarditis Presenting as Leukocytoclastic Vasculitis

**DOI:** 10.7759/cureus.1464

**Published:** 2017-07-13

**Authors:** Sarah El Chami, Atieh Jibbe, Shadi Shahouri

**Affiliations:** 1 Internal Medicine, University of Kansas School of Medicine - Wichita; 2 Internal medicine/ Rheumatology, Arthritis and Rheumatology Clinics of Kansas

**Keywords:** vasculitis, endocarditis, cutaneous, immunosuppression

## Abstract

Subacute bacterial endocarditis can have many different presentations; in rare instances, it can present as leukocytoclastic vasculitis owing to the effect of circulating immune complexes and micro-emboli on the vascular endothelium. A high index of suspicion needs to be maintained to differentiate between infectious vs noninfectious autoimmune vasculitides, keeping in mind that missing a diagnosis can have fatal results. In this case report, we introduce a young female patient who initially presented with a picture of idiopathic autoimmune cutaneous vasculitis delaying the diagnosis of an underlying infective endocarditis with aortic valve involvement.

## Introduction

Cutaneous leukocytoclastic vasculitis can be a presenting feature in multiple different medical conditions. This can be problematic when directly competing etiologies are in the differential. Empiric treatments may potentially harm a misdiagnosed patient, for e.g., providing immunosuppression may prove fatal if the true etiology is infectious. This is a case of a patient who presented initially with cutaneous leukocytoclastic vasculitis of unclear etiology and was later diagnosed with bacterial endocarditis. We further discuss previously reported cases of cutaneous leukocytoclastic vasculitis, paying close attention to features that would suggest an infectious versus an autoimmune etiology.

## Case presentation

**Figure 1:**
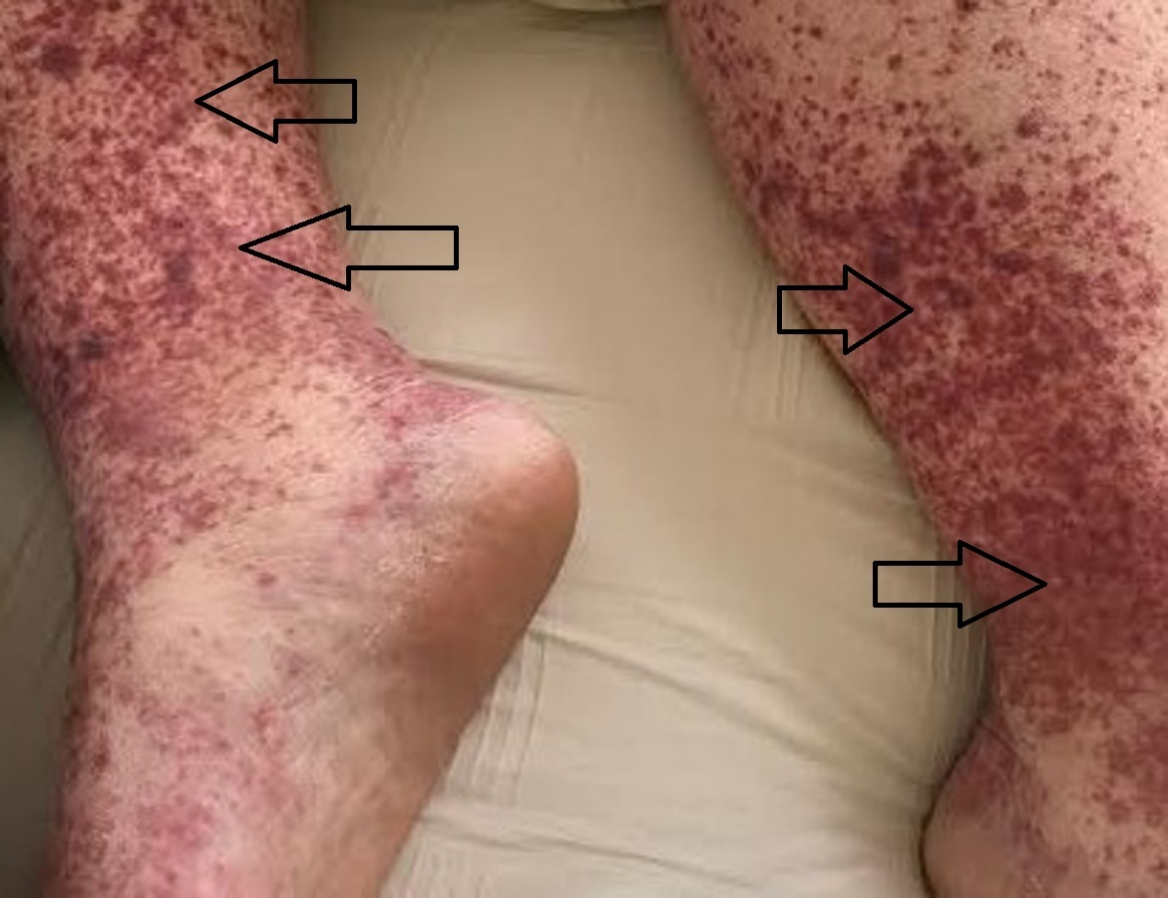
Bilateral view of pigmented purpuric macules coalescing into purpuric plaques on lower extremities bilaterally

**Figure 2:**
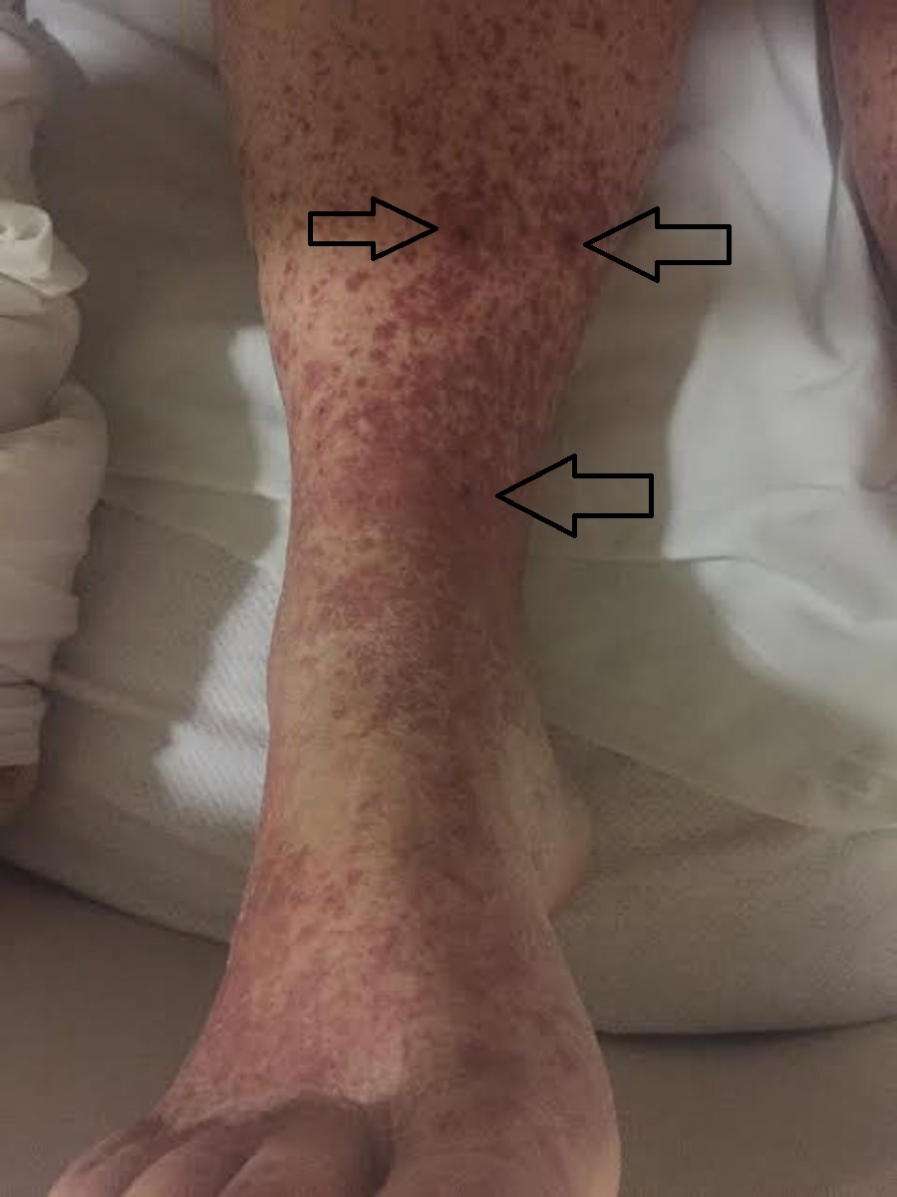
Unilateral view of pigmented purpuric macules coalescing into purpuric plaques on lower extremities bilaterally

A 42-year-old female patient presented initially to her obstetric and gynecologist with a non-blanching purpuric rash involving bilateral lower extremities (Figures 1-2). Initial lab results revealed anemia with hemoglobin of 8.9 g/d and she was started on iron supplements. The rash was thought to be secondary to citalopram and, at the time, was switched to sertraline. Her past medical history was significant for anxiety, hypothyroidism, and a recent premature cesarean section for five months due to pre-eclampsia. This was complicated by wound dehiscence and infection requiring debridement with negative pressure wound therapy.

Ten days later, she presented for night sweats. The lab results were remarkable for a drop in hemoglobin from 8.9 g/dl to 6.4 g/dl. She was admitted to a tertiary center a day later for fever and lethargy. Her body temperature in the emergency department was 38.4 degrees Celsius, heart rate 101bpm, blood pressure 90/40, and O2 saturation 92%. She was worked up for acute hemolytic anemia and purpura.

Lab results revealed white blood cell count 7.3 with 82% neutrophils, 8% bands and toxic granulation, hemoglobin 8.3 g/dL after two units pack red blood cells, platelets 192 k/cumm, prothrombin time (PT) 1.2 sec, partial prothrombin time (PTT) 26 sec, lactic acid 1.1 mmol/L, absolute retic count 62 k/cumm, retic 1.9%, haptoglobin 256 mg/dL, lactate dehydrogenase 294 units/L, erythrocyte sedimentation rate 45 mm/hr, c-reactive protein 123 mg/L, and creatinine 1.1 mg/dL. A peripheral smear showed microcytic hypochromic anemia, anisochromia, anisopoikilocytosis, and nonspecific absolute lymphocytopenia. Urinalysis showed trace protein with 4+ blood, 0-3 red blood cells, 4+ bacteuria, hyaline and granular casts, negative nitrite, and leukocyte esterase. Urine protein/ creatinine ratio was 621mg/g, creatinine kinase 29 units/L, C3 63 mg/dL (90-189), and C4 <2 mg/dL (10-40). Of note, cryoglobulins were positive while the following labs were negative: hepatitis panel, human immunodeficiency virus (HIV), anti-nuclear antibody (ANA), Anti-phospholipids Abs, anti-glomerular basement membrane (anti-GBM), cytoplasmic/peripheral anti-neutrophil cytoplasmic antibodies (c/p ANCA), and anti-myeloperoxidase. However, anti-proteinase was positive at 3.6 U/mL (0-3.5). A skin biopsy showed leukocytoclastic vasculitis. The patient was thought to have Henoch-Schonlein Purpura (HSP) or ANCA-associated vasculitis but immunosuppressive therapy was delayed and antibiotics were initiated after blood cultures came back positive for Enterococcus faecalis. Her condition was deteriorating; she had hypothermia, tachycardia, worsening anemia, and developed respiratory failure requiring intubation. A trans-esophageal echocardiogram (TEE) revealed a large 1.6 cm vegetation on the aortic valve with severe aortic insufficiency though there was no correlating murmur or other cardiac findings on physical exam. The aortic valve was destroyed with dissection into the left ventricle; pathology showed extensive degenerated and necrotic endocardial tissue with evidence of endocarditis. Her clinical status including the severity of the rash improved after the aortic valve (AV) replacement and she was successfully discharged from the hospital.

## Discussion

The presentation of endocarditis with a cutaneous vasculitis has been well reported. In fact, we argue that endocarditis should be placed on a physician’s differential when manifestations of vasculitis are seen in a septic patient. In a 2015 study published in Clinical and Experimental Rheumatology, researchers assessed the presentation of severe bacterial infection with the clinical manifestation of cutaneous vasculitis. Out of the studied 766 patients presenting with cutaneous vasculitis, 27 patients were diagnosed with an underlying bacterial infection and six of the 27 were diagnosed with endocarditis as the cause of the bacterial infection [1].

Another case was published in the Clinical Medicine Journal in 2012. The patient presented with a vasculitic rash, anemia, and positive dipstick for protein and blood. The initial diagnosis was HSP, as was in our case, until blood cultures from admission came back positive [2].

Another example of this was described in the Journal of The American Society of Nephrology. The patient presented with a vasculitic rash and renal failure and was diagnosed with essential type III cryoglobulinemia. Despite initial improvement with immunosuppressive therapy, he deteriorated six weeks later leading to the diagnosis of subacute bacterial endocarditis and dramatically improved with antibiotics. This indicates that temporary improvement after treatment does not confirm the diagnosis [3]. Similarly, our patient also tested positive for cryoglobulins, had minor renal involvement and a vasculitic rash.

Endocarditis can sometimes mimic ANCA vasculitis as well. A few cases of bacterial endocarditis presenting as ANCA vasculitis with positive ANCA were described in the literature. In 2015, a case was published in the Internal Medicine Journal-Tokyo describing a patient with rapidly progressive glomerulonephritis (RPGN) associated with subacute bacterial endocarditis (SBE) due to *Enterococcus faecalis* infection. Similar to our patient, that patient exhibited positivity for anti-proteinase three-antineutrophil cytoplasmic antibody (PR3-ANCA) and had low complement levels; he also tested positive for rheumatoid factor (RF).  RPGN improved, both PR3-ANCA and RF were undetectable after antibiotics and valve replacement [4].

Another case was described in the Journal of UOEH in 2010 where a patient presenting with fatigue and fever was initially treated for pneumonia. He remained febrile despite antibiotics and further work-up revealed high myeloperoxidase specific ANCA titer (MPO-ANCA); steroid pulse therapy was started with initial improvement followed by severe deterioration and death. Autopsy revealed bacterial infective aortic valve vegetation with infected thrombo-emboli and micro-abscesses in many organs [5].

Immunosuppression is the gold standard treatment for most types of vasculitis, however misdiagnosing a case of bacterial endocarditis and initiating immunosuppressive therapy can lead to detrimental outcomes as in the aforementioned case.

On the other hand, many rheumatologic diseases can mimic infective endocarditis in a similar fashion. In 2003, a case was described in the Italian Heart Journal of a patient with known mitral valve prolapse and moderate regurgitation presenting with fever of unknown origin and misdiagnosed as infective endocarditis, after further investigations, he was found to have Wegner's granulomatosis [6].

It remains crucial to differentiate the two different sets of diseases to avoid catastrophic outcomes, keeping in mind the overlapping constellation of symptoms and diagnostic markers.

## Conclusions

In conclusion, it becomes clear that cutaneous vasculitis is a rare though potential presentation of bacterial endocarditis. The initial presentation of the latter can be misleading and a high index of suspicion needs to be maintained to avoid adverse and sometimes fatal outcomes derived from providing the wrong treatment. It is also crucial not to be fooled by the initial response to treatment in confirming the diagnosis in such patients.
